# Research on an evaluation index system and evaluation method of green and low-carbon expressway construction

**DOI:** 10.1371/journal.pone.0283559

**Published:** 2023-03-27

**Authors:** Haiying Wang, Jianyu Tao, Jian Xu, Yingzhi Zhang

**Affiliations:** 1 School of Construction Machinery, Chang’an University, Xi’an, Shaanxi, China; 2 Xi’an University of Science and Technology, Xi’an, Shaanxi, China; 3 Shaanxi Transportation Holding Group Co., Ltd., Xi’an, Shaanxi, China; Shandong University of Science and Technology, CHINA

## Abstract

To promote the development of green and low-carbon expressway construction, an evaluation index system and associated evaluation method for the construction of green and low-carbon expressways suitable for multiple bridges and tunnels is proposed. The evaluation index system was created from three layers: the goal layer, the criterion layer, and the indicator layer. The criterion layer includes 4 first-level indices and the indicator layer includes 18 second-level indices. The weight of each index in the criterion layer and indicator layer is determined using the improved analytic hierarchy process (AHP) method, and green and low-carbon expressway construction is then graded using the gray fuzzy comprehensive evaluation method combined quantitative indices with qualitative indices. The method with the selected indices is then verified in a case study on the Huangling-Yan’an Expressway, with the evaluation grade being “Excellent” and the evaluation value being 9.1255. The proposed evaluation method can provide theoretical and practical guidance for effective evaluation for green and low-carbon expressway construction.

## 1 Introduction

By the end of 2021, the total length of the highway in China reached 5.28 million kilometers, of which the length of the expressway reached 0.169 million kilometers. The huge consumption of energy and the emission of pollutants in the process of expressway construction have caused great damage to the ecological environment [1, 2]. With the intensification of global environmental and energy problems, sustainable development, energy conservation and emission reduction have attracted increasing attention. Therefore, exploring a green and low-carbon expressway construction model has become the primary task of the construction industry.

Relatively mature green highway evaluation systems have been developed more than ten years ago. A voluntary sustainability rating system “Greenroads”, established by University of Washington and CH2M Hill in 2009, is composed of five aspects: Construction Activities, Materials & Design, Utilities & Controls, Access & Livability, and Creativity & Effort [3–5]. The “Greenpave” is a road sustainability rating system developed by the Ontario Ministry of Transportation in 2011, which defines specific strategies and goals that contribute to road sustainability, primarily on pavement components and pavement-related projects including long life pavements, recycled content and other 14 items [6, 7]. In 2012, the U.S. Federal Highway Administration developed the Infrastructure Voluntary Evaluation Sustainability Tool (INVEST) system with support from CH2M Hill. It is a free, web-based tool that covers the complete multiple phases of transportation services [7, 8].

In China, sustainable development was regarded as an important national development strategy at the 15^th^ National Congress of the Communist Party of China in 1997. In recent years, the construction of green highway and its related evaluation methods have developed rapidly. In 2016, the Ministry of Transport of China issued *Guidance on the implementation of green highway construction*, which put forward the main tasks and goals of green highway development [9]. In 2018, the Technical Committee on Transport Environmental Protection Standardization of China issued *Technical requirements for the assessment of green transportation facilities -Part1: Green highway*, stipulating the basic requirements, evaluation index system and methods for green highway evaluation [10]. In 2020, the Ministry of Transport of China issued *Comprehensively summarize practical experience and promote the transformation and upgrading of green highway construction*, which proposed to create a number of pilot projects for green highway construction, focusing on highway reconstruction, expansion, construction in ecologically sensitive and vulnerable areas, and the construction of major national strategic corridors [11]. In 2022, the Ministry of Transport of China issued *The 14^th^ Five-Year development plan of highways*, pointing out that it is necessary to continue to deepen the construction of green roads, strengthen energy conservation, emission reduction and pollution prevention and control in the field of road transportation, and comprehensively improve the level of green development of the highway industry [12].

In summary, the main problems on the evaluation index systems and evaluation methods of green and low-carbon expressway are: (1) The currently used *Green Cycle Low-Carbon Highway Assessment and Evaluation Index System (Trial)* (hereinafter called *Standard*) quantify the score of each index, but some qualitative indices (such as energy conservation and emission reduction organization and working mechanism, publicity and training, etc.) are often scored by yes or no, and the implementation of some indices is not specifically quantified; (2) Most of the evaluation systems focus on the whole life cycle of green highway, and little consideration is given to the green and low-carbon indices of the construction period with the most pollution emissions; (3) The same set of index system is used for both highways and expressways, and little consideration is given to the characteristics of multiple bridges and tunnels of expressways. Therefore, it is of great theoretical and practical significance to propose a set of green and low-carbon evaluation index system and corresponding evaluation methods during expressway construction, focusing on the expressway characteristics multiple tunnels and bridges.

## 2 Literature review

### 2.1 Evaluation indices

Li [13] et al. established the concept of green ecological urban roads, put forward five first-level indices including design rationality, road function effect, etc., and comprehensively evaluated green ecological roads. Zhang [14] et al. established a green highway evaluation index system from seven aspects including green concept, ecological protection, etc., and gave the score conversion method after removing the non-participation indicators. Tu [15] et al. divided the green highway assessment into three parts from the perspective of process control and management: planning & design, construction, and operation & maintenance, and constructed three first-level indices of green design, green construction and green maintenance accordingly. Based on the PSR framework model and combined with the target decomposition method, Li [16] et al. constructed the green highway evaluation index system of Jiangxi Province from five aspects, including green concept, ecological environmental protection, etc. Xie [17] et al. combined with the undulations of terrain and the requirements of expressway construction to construct an index system for evaluating the carrying capacity of resources and environment for expressway construction.

Specific indices of different index systems in some of the literature are shown in Table 1.

**Table 1:** Specific indices of different index systems in some of the literature.

First-level indices	Further descriptions (second and third-level indices)	References
Two modules: required and voluntary credits.	Required credits must be completed to obtain credits, and voluntary credits include five aspects: Construction Activities, Materials & Design, Utilities & Controls, Access & Livability, Creativity & Effort.	Greenroads [5]
Four modules: System planning for states, system planning for regions, Operations & Maintenance, and Project Development.	The Project Development module consists of multiple voluntary indices from 3 aspects: road network planning, project design & construction, and project operation management..	INVEST [7, 8]
Five indices: design rationality, road function effect, energy saving & emission reduction effect, greening effect, environmental protection effect.	The various green road effects in the first-level indices are subdivided into 14 second-level indices and 31 third-level indices.	Literature [13]
Seven indices: green concept, ecological protection, resource conservation, energy conservation & low carbon, safety & wisdom, service improvement, and quality construction.	Each first-level index is expanded to second-level indices according to its content, and there are 22 second-level in-dices such as soil and water environment protection, material conservation and recycling, etc., and 58 third-level indices.	Literature [14]
Three indices: green design, green construction and green maintenance.	Each first-level indices consists of control items and scoring items, among which the control items are the basic requirements for green roads, and the results are only satisfied or not satisfied, and the scoring items are the main basis for rating evaluation grades, with 40 second-level indices in total.	Literature [15]
Five indices: green concept, ecological environmental protection, resource conservation, energy conservation & low carbon, smart service.	The first-level indices are subdivided into 9 categories, including management & culture, ecological landscape, and natural resource conservation, etc. The 9 categories are further divided into 18 second-level indices, and 52 third-level indices according to the scoring standards.	Literature [16]

The main problems in the current green highway evaluation index systems are: (1) There are too many qualitative indices in evaluation index system, resulting in the subjectivity of the evaluation results; (2) The number and complexity of indices are too large, which is not conducive to obtaining evaluation results conveniently, and the generality of the complex index system is poor.

### 2.2 Evaluation methods

For the comprehensive evaluation of multi-index system, the weight of index is generally determined first, and then the fuzzy comprehensive evaluation method is used for calculation [18–21]. The determination of index weight commonly adopts AHP [22–24], Delphi method [25], entropy weight method [26, 27], etc.

In terms of green and low-carbon highway evaluation, Yang [28] proposed that the evaluation system from different perspectives can be different, and the weight analysis for indices based on facility layout was carried out by the entropy method, while weight analysis for indices based on user perception was suitable to use AHP method. Chen [29] used AHP-fuzzy comprehensive method to evaluate the effect of highway greening, and its membership function is obtained by experts scoring and counting the proportion of scorers, which is highly subjective. Zhang [30] et al. used the AHP-fuzzy comprehensive evaluation method to evaluate the environmental impact of rural roads during construction, and characterized the consistency and credibility of expert scores by introducing the mean and standard deviation of the score values. Zhao [31] et al. used the fuzzy hierarchy comprehensive evaluation method to assess the risk of long-steep downgrade sections of expressways, and the set value statistical method and the linear standard method are used for qualitative and quantitative indicators respectively to make all indicators be quantitatively evaluated together. Bai [32] et al. combined the AHP and entropy method to determine the weight of each index, and evaluated the regional rural highway construction and development by set pair analysis method. Shi [33] et al. established a gray clustering model and performed hierarchical clustering analysis of the Yichun-Wudalianchi highway in seasonal frozen zone. Wang [34] et al. used the C-OWA operator to calculate the weight of each index, and used the gray clustering method to construct the green construction evaluation model for desert highways. He [35] et al. established an extensible cloud model with matter element analysis theory and cloud model to evaluate the green ecology of municipal roads and realized visual cloud image analysis. Nusa [36] et al. used Partial Least Squares—Structural Equation Modeling to relate the driving factors of characteristics and challenges of green highway with the success factors to implement green highway in Malaysia. Kokkaew [37] et al. proposed a framework of the green assessment called Green Growth Index to combine project environmental index and economic into a single index that can be easily used by project managers.

The main problems in the evaluation methods of green highway are: (1) The widely used AHP method is easily affected by the wrong judgment of some experts; (2) For qualitative indicators, too much reliance on expert scoring and lack of mathematical calculation integration; (3) Lack of comprehensive evaluation methods for quantitative and qualitative indices.

In summary, a set of evaluation index system for green and low-carbon expressway construction on the basis of the Standard is proposed in this paper, focusing on the stage of expressway construction and combining the characteristics of multiple tunnels and bridges of expressways. The improved AHP method is used to calculate the weight of each index. The fusion of quantitative and qualitative indices is realized by the same fuzzy membership function and whitening weight function, and comprehensive evaluation is carried out by gray fuzzy comprehensive evaluation method. This evaluation system improves the defects that the values of some indices in the Standard are not clear enough, the weights of each index are the same, and quantitative and qualitative indices cannot be comprehensively evaluated in traditional evaluation methods. It is applicable to the green and low-carbon evaluation of expressway construction with different characteristics.

## 3 Evaluation index system and evaluation methods

### 3.1 Establishment of evaluation index system

The construction of green and low-carbon expressway is a multi-level and multi-objective system involving many fields such as construction energy consumption control, resource utilization technology, construction technology, project management, and construction area characteristics. The establishment of the evaluation system needs to consider the various fields involved comprehensively, and take the accuracy, objectivity, comprehensiveness, and operability of the evaluation indices as the principle.

**Fig 1:**
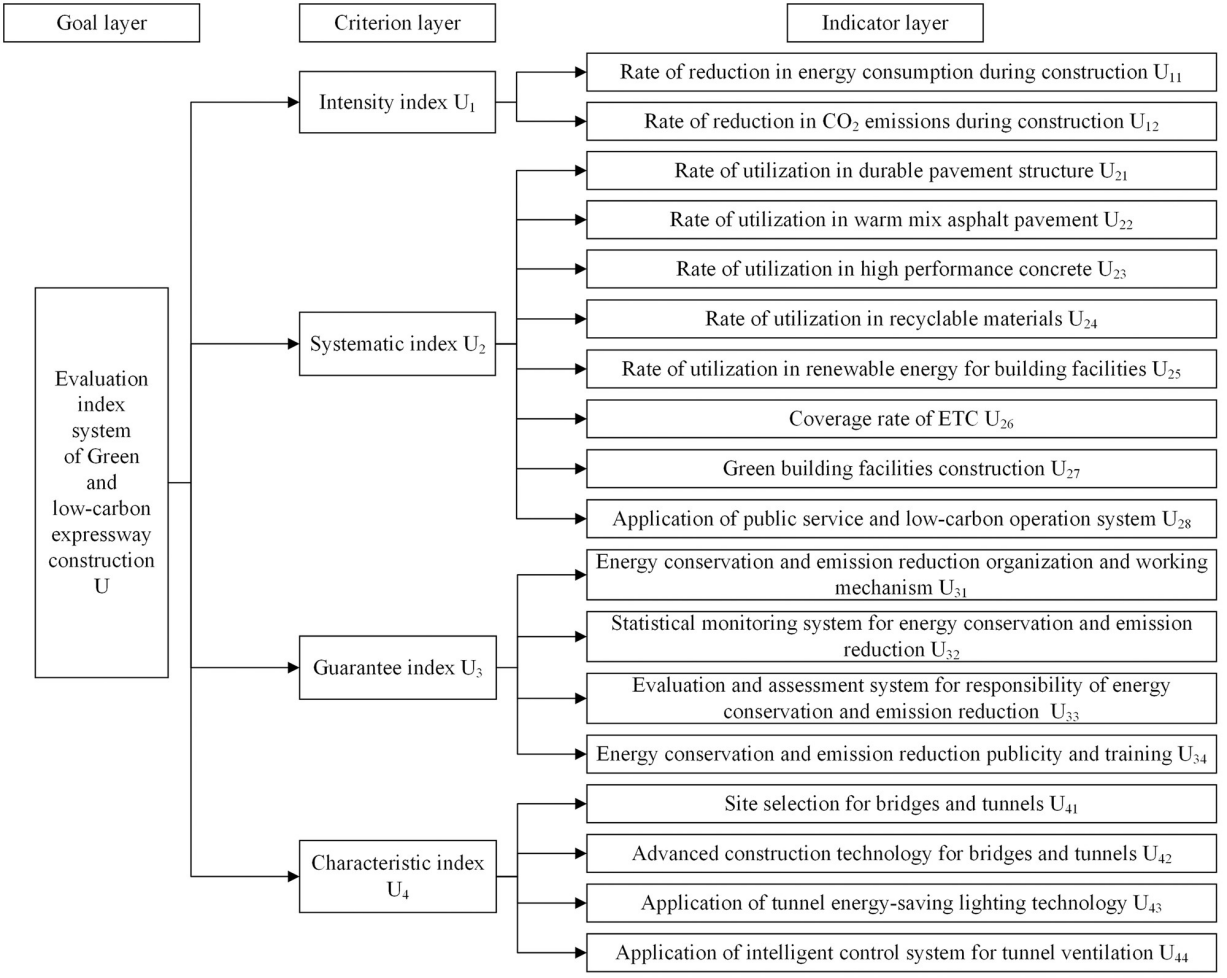
The proposed green and low-carbon expressway construction evaluation index system.

On the basis of *Standard*, relevant indices such as expressway bridges and tunnels are added to the evaluation index system as characteristic indices. The evaluation index system is divided into the goal layer, the criterion layer, and the indicator layer. The goal layer is the green and low-carbon expressway construction evaluation index system. The criterion layer includes four primary indices: intensity index, systematic index, guarantee index, and characteristic index. The indicator layer belongs to the secondary index layer, with a total of 18 secondary indices, including two intensity indices, eight systematic indices, four guarantee indices and four characteristic indices, as shown in Fig 1.

Referring to Table 1, the advantages of the proposed index system are as follows: (1) Compared with the index system of the whole life cycle, the proposed index system focuses on the construction period of expressway, and the summary of the construction period indices is more adequate; (2) The introduction of characteristic indices can make green and low-carbon evaluation of expressways with different geomorphic features more flexible; (3) The proposed index system with more quantitative indicators can make evaluation results more objective and accurate.

### 3.2 Determine the weights of indices using the improved AHP method

In the proposed evaluation system, each evaluation index is given a different weight in the minds of decision makers. The main difficulty in determining the weight of each evaluation index is that these weights are often not easy to quantify. Where there are multiple sub-factors affecting a factor, when directly considering how much weight each factor has on that factor, it is often possible for decision makers to present weights that are inconsistent with their actual importance due to incomplete consideration.

The AHP method is used to solve the qualitative and quantitative combination of multi-objective problems of the decision analysis method. AHP uses the empirical judgment of decision makers to measure the relative importance between the criteria, and gives the weight of each criterion. As AHP is more effective in solving the problem that is difficult to solve by quantitative methods, it is applicable to the multi-level, multi-factor, and quantitative indices combined with qualitative indices of the evaluation system of green and low-carbon expressway construction. To solve the problem that traditional AHP method is susceptible to misjudgment by some experts, an improved AHP method is proposed to determine the index weights of the proposed green and low-carbon expressway construction evaluation index system.

**Fig 2:**
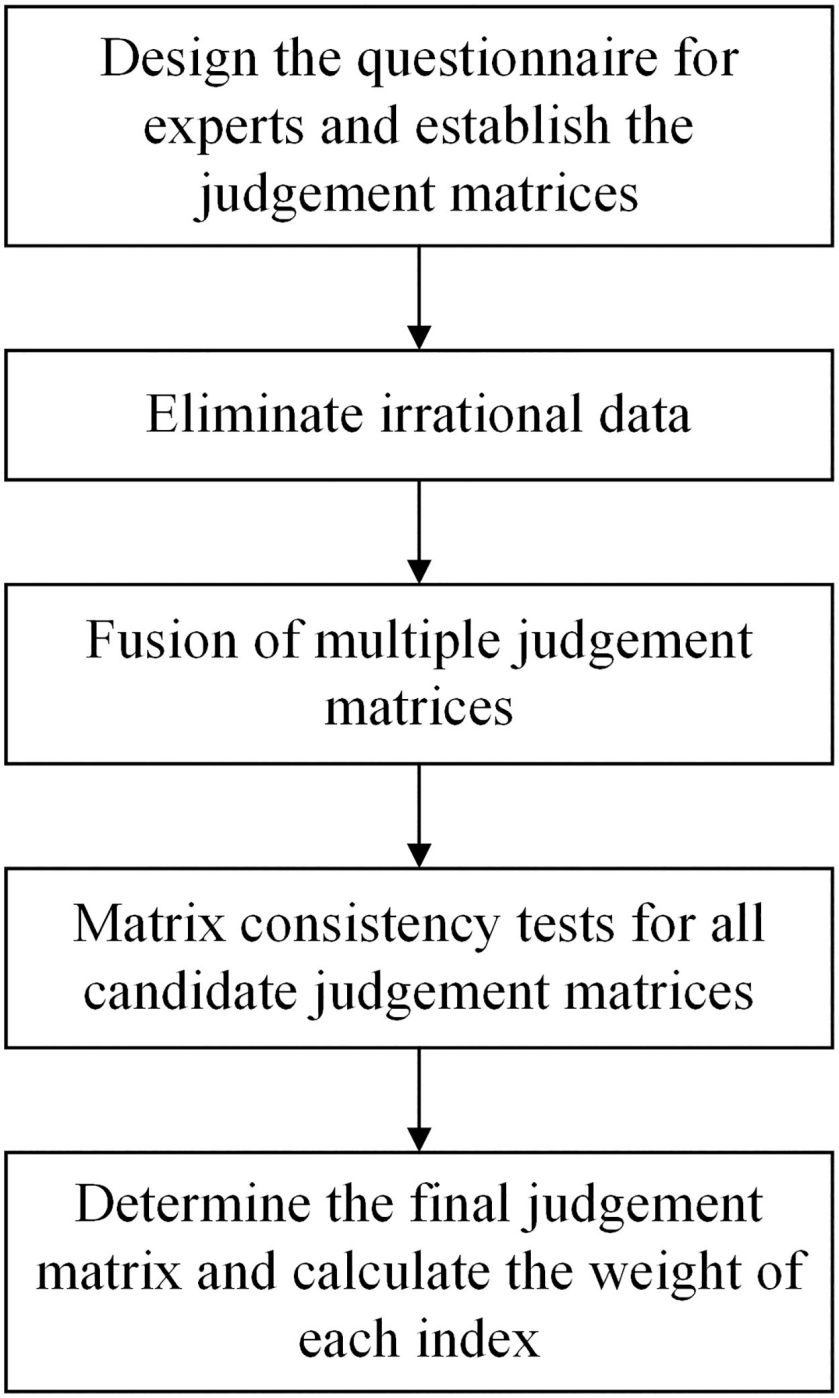
The roadmap of the improved AHP method.

The roadmap of the improved AHP method is shown in Fig 2.

Step 1: Establish the judgment matrices

To ensure the objectivity and professionalism of the weights obtained, a questionnaire was designed to compare the evaluation index at the same level in the green and low-carbon expressway evaluation system based on a rating scale of 1–9 (as shown in Table 2). The questionnaire was distributed to the invited evaluation experts, on-site construction engineers, and highway construction project managers. According to the degree of importance, the value of each index was assigned by the experts, and the judgment matrices were obtained. Take the indices in criterion layer as an example, the judgment matrix example is shown in Table 3.

**Table 2:** Green and low-carbon expressway evaluation system rating scale.

Digital scale	Definition
1	The two elements are equally important
3	One element is a little more important than the other
5	One element is more important than the other
7	One element is much more important than the other
9	One element is absolutely more important than the other
2, 4, 6, 8	Values associated with intermediate judgments

**Table 3:** Judgment matrix of the criterion layer index.

	*U*_1_	*U*_2_	*U*_3_	*U*_4_
*U*_1_	1	*a*_12_	*a*_13_	*a*_14_
*U*_2_	1/*a*_12_	1	*a*_23_	*a*_24_
*U*_3_	1/*a*_13_	1/*a*_23_	1	*a*_34_
*U*_4_	1/*a*_14_	1/*a*_24_	1/*a*_34_	1

Step 2: Eliminate irrational data

Calculate the median of the corresponding elements of each judgment matrix, and eliminate irrational data that conform to the following conditions:

(1) (median−1) × (corresponding element data−1)< 0;

(2) |median−corresponding element data| > 2.

Step 3: Fusion of multiple judgment matrices

The average values of the corresponding elements of each judgment matrix after eliminating irrational data is calculated and consist of a new matrix. Find the element in the new matrix greater than or equal to 1. If it is an integer, it will be used as the corresponding element of final judgment matrix and the element with respect to the main diagonal symmetry is taken as the reciprocal; if it is not an integer, the value is rounded up and down respectively to obtain two candidate elements and the element with respect to the main diagonal symmetry is respectively taken as the reciprocal of the two candidate elements. Combine all candidate elements in pairs to obtain multiple candidate judgment matrices.

Step 4: Matrix consistency tests for all candidate judgment matrices

The matrix consistency test is shown in Eqs (1) and (2).

Calculate the coherence index CI and the random consistency ratio CR.

$$\begin{array}{c} CI=\frac{\lambda_{max}-n}{n-1} \end{array}$$ (1)

$$\begin{array}{c} CR=\frac{CI}{RI} \end{array}$$ (2)

Where:

λ_*max*_–the maximum eigenvalue of the judgment matrix;

*n*–the order of judgment matrix;

*RI*–Average random consistency index, the values are shown in Table 4.

**Table 4:** Judgment matrix of the criterion layer index.

*n*	12	2	3	4	5	6	7	8	9	10
*RI*	0.00	0.00	0.58	0.90	1.12	1.24	1.32	1.41	1.45	1.49

In general, if *CR* ≤ 0.1, the judgment matrix is considered consistent and the weight can be calculated; otherwise, the judgment matrix needs to be adjusted.

Step 5: Determine the final judgment matrix and calculate the weight of each index

The judgment matrix with the smallest *CR* value is selected as the final judgment matrix. The index weight vector, *W*, is obtained by normalizing the eigenvector corresponding to the maximum eigenvalue, λ_*max*_, of the final judgment matrix. The elements of *W* represent the relative importance of the same level index to the higher level.

### 3.3 Gray fuzzy comprehensive evaluation method

The gray fuzzy comprehensive evaluation method is an organic combination of the gray evaluation method and the fuzzy comprehensive evaluation method. The advantage of this method is that it cannot only deal with the gray problem—which has the characteristics of uncertainty, poor information, and small sample size—but also solve the problem by analyzing the impact of relevant factors on the problem in a fuzzy environment, and make a comprehensive evaluation based on the fuzzy mathematical methods.

**Fig 3:**
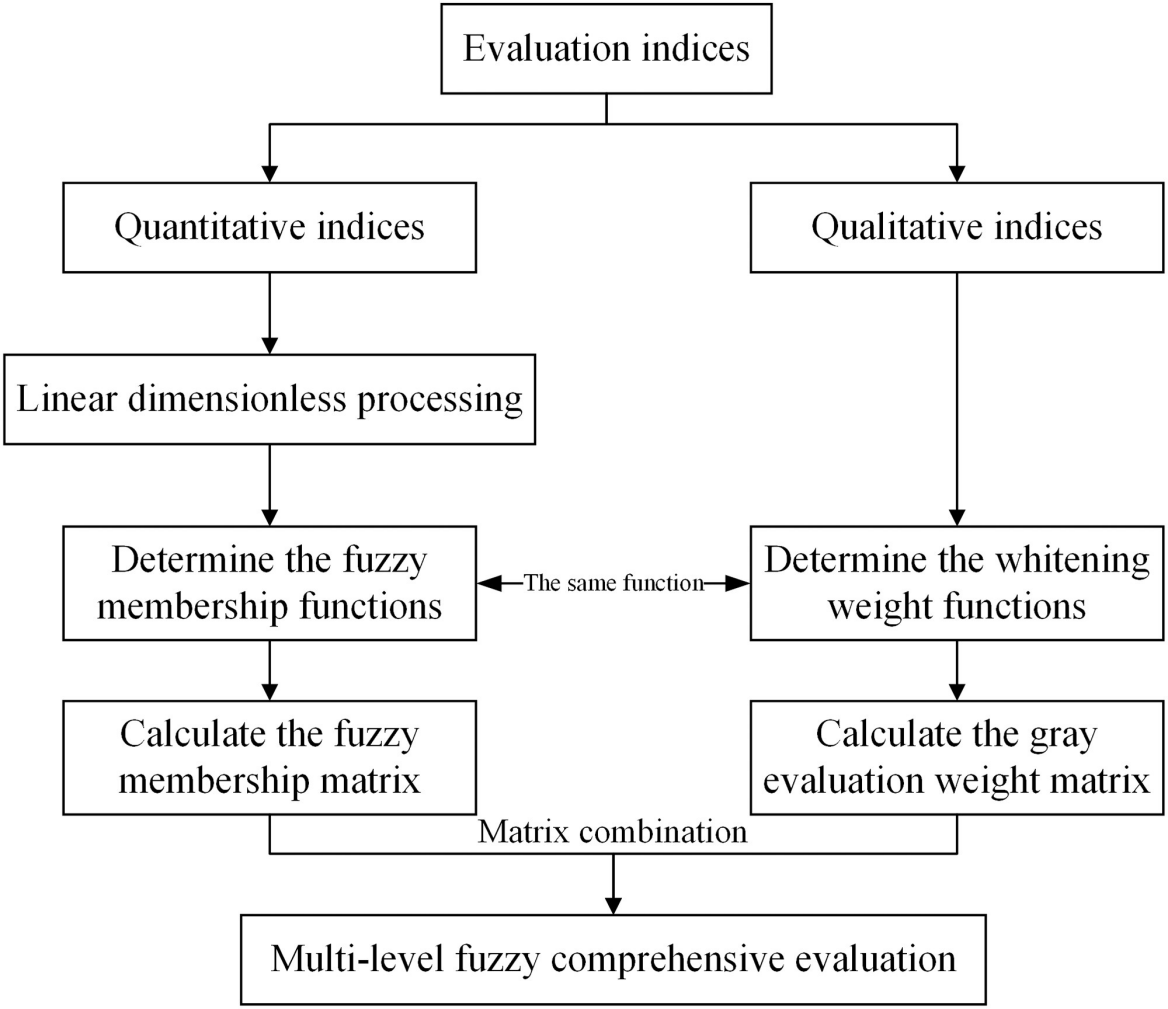
The roadmap of gray fuzzy comprehensive evaluation method.

The roadmap of gray fuzzy comprehensive evaluation method is shown in Fig 3.

#### 3.3.1 Linear dimensionless processing of quantitative indices

The green and low-carbon expressway construction evaluation indices in the indicator layer are divided into quantitative indices and qualitative indices according to the index properties. A linear dimensionless method was used to preprocess quantitative indices *U*_*ij*_ in the indicator layer. Assume the calculated value of the index *U*_*ij*_ to be *u*_*ij*_ and the defined interval of each index to be [*A*_*ij*_, *B*_*ij*_], where *B*_*ij*_ > *A*_*ij*_ > 0, as shown in Table 5. The quantitative processing result using Eq (3) for *u*_*ij*_ is *x*_*ij*_ ∈ [0, 10], a dimensionless numeric value.

$$\begin{array}{c} x_{ij}=\{\begin{array}{cc} 10, & u_{ij}\geq B_{ij}\\ 10\times \frac{u_{ij}-A_{ij}}{B_{ij}-A_{ij}}, & u_{ij}\in \left(A_{ij},B_{ij}\right)\\ 0, & u_{ij}\leq A_{ij} \end{array} \end{array}$$ (3)

**Table 5:** The defined intervals of each quantitative secondary index.

Quantitative indices in indicator layer	*A*_*ij*_	*B*_*ij*_	Calculation method(×100%)
Rate of reduction in energy consumption during construction *U*_11_	20%	40%	$\frac{Reduction\;in\;energy\;consumption}{Energy\;consumption\;of\;conventional\;schemes}$
Rate of reduction in CO_2_ emissions during construction *U*_12_	20%	30%	$\frac{Reduction\;in\;CO_{2}\;emissions}{CO_{2}\;emissions\;of\;conventional\;schemes}$
Rate of utilization in durable pavement structure *U*_21_	0%	10%	$\frac{The\;area\;of\;durable\;pavement}{The\;area\;of\;total\;pavement}$
Rate of utilization in warm mix asphalt pavement *U*_22_	0%	10%	$\frac{The\;area\;of\;warm\;mix\;asphalt\;pavement}{The\;area\;of\;total\;warm\;mix\;asphalt\;pavement}$
Rate of utilization high performance concrete *U*_23_	0%	50%	$\frac{High\;performance\;concrete\;usage}{Total\;concrete\;usage}$
Rate of utilization in recyclable materials *U*_24_	0%	50%	$\frac{Recyclable\;materials\;usage}{Planned\;raw\;materials\;usage}$
Rate of utilization in renewable energy for building facilities *U*_25_	0%	15%	$\frac{Number\;of\;facilities\;applying\;renewable\;energy}{Total\;number\;of\;facilities}$
Coverage rate of ETC *U*_26_	0%	60%	$\frac{The\;number\;of\;ETC\;stations}{The\;number\;of\;total\;toll\;stations}$

#### 3.3.2 Fuzzy membership of quantitative indices

The calculated values of all quantitative indices in the indicator layer were grouped into a factor set $X=\{x_{1},x_{2},\cdots ,x_{n_{1}}\}$ in the order in which they appeared in the indicator layer. *n*_1_ represents the number of quantitative indices in the indicator layer. The comment set is set as {*V*_1_, *V*_2_, ⋯, *V*_*k*_, ⋯, *V*_*m*_} (generally the qualitative descriptive statement), with the quantitative criteria for each evaluation grade *V* = {*v*_1_, *v*_2_, ⋯, *v*_*k*_, ⋯, *v*_*m*_}. The semi-trapezoidal and triangular membership functions are used to determine the fuzzy membership of the index, that is, by dividing the demarcation point on the continuous interval, the membership is obtained by linear interpolation according to the size of index value. The membership function is shown as follows:

$$\begin{array}{c} f_{1}\left(x_{i}\right)=\{\begin{array}{cc} 1, & x_{i}<v_{1}\\ \frac{v_{2}-x_{i}}{v_{2}-v_{1}}, & v_{1}\leq x_{i}<v_{2}\\ 0, & x_{i}\geq v_{2} \end{array} \end{array}$$ (4)

$$\begin{array}{c} f_{2}\left(x_{i}\right)=\{\begin{array}{cc} 1-f_{1}\left(x_{i}\right), & v_{1}<x_{i}<v_{2}\\ \frac{v_{3}-x_{i}}{v_{3}-v_{2}}, & v_{2}\leq x_{i}<v_{3}\\ 0, & x_{i}\geq v_{1}\;or\;x_{i}\geq v_{3} \end{array} \end{array}$$ (5)

$$\begin{array}{c} \vdots \end{array}$$ (6)

$$\begin{array}{c} f_{k}\left(x_{i}\right)=\{\begin{array}{cc} 1-f_{k-1}\left(x_{i}\right), & v_{k-1}<x_{i}<v_{k}\\ \frac{v_{k+1}-x_{i}}{v_{k+1}-v_{k}}, & v_{k}\leq x_{i}<v_{k+1}\\ 0, & x_{i}\geq v_{k-1}\;or\;x_{i}\geq v_{k+1} \end{array} \end{array}$$ (7)

$$\begin{array}{c} \vdots \end{array}$$ (8)

$$\begin{array}{c} f_{m}\left(x_{i}\right)=\{\begin{array}{cc} 1-f_{m-1}\left(x_{i}\right), & v_{m-1}<x_{i}<v_{m}\\ 1, & x_{i}\geq v_{m} \end{array} \end{array}$$ (9)

According to Eqs (4)–(9), the fuzzy membership vector of the quantitative evaluation index value *x*_*i*_ for evaluation grade *v*_*k*_ is obtained, and all the fuzzy membership vectors constitute the fuzzy membership matrix *R*_1_.

#### 3.3.3 Gray evaluation weight of qualitative indices

For qualitative indices in the indicator layer, the gray evaluation weight matrix is determined by combining expert scoring with the whitening weight function in gray theory, and the steps are as follows:

Step 1: Establish the evaluation sample matrix using expert scoring method

An expert group composed of *p* experts evaluated and scored (based on 10-point scoring) the *n*_2_ qualitative secondary indices in green and low-carbon expressway evaluation index system. The score given by the *i*^*th*^ expert on the *j*^*th*^ evaluation index was recorded as *h*_*ij*_, and the evaluation sample matrix H composed of *h*_*ij*_ can be expressed as:

$$\begin{array}{c} H=[\begin{array}{cccc} h_{11} & h_{12} & \cdots & h_{1n_{2}}\\ h_{21} & h_{22} & \cdots & h_{2n_{2}}\\ \cdots & \cdots & \cdots & \cdots \\ h_{p1} & h_{p2} & \cdots & h_{pn_{2}} \end{array}] \end{array}$$

Step 2: Determine the evaluation gray classes and whitening weight functions

Considering that the gray evaluation weight matrix obtained in this section needs to be combined with the fuzzy membership matrix obtained in Section 3.3.1, the evaluation gray classes and whitening weight functions should be consistent with the comment set and fuzzy membership function in Section 3.3.1 respectively. The quantified vector for each evaluation gray class is *G* = *V* = {*v*_1_, *v*_2_, ⋯, *v*_*k*_, ⋯, *v*_*m*_}.

Step 3: Calculate the gray evaluation coefficient

Calculate the weight *f*_*k*_(*h*_*ij*_) for *h*_*ij*_ belonging to the *k*^*th*^ evaluation grade, and calculate the gray evaluation coefficient *n*_*jk*_ of the *j*^*th*^ qualitative index for the *k*^*th*^ evaluation grade and the total gray evaluation coefficient *n*_*j*_ of the *j*^*th*^ qualitative index using Eqs (10) and (11).

$$\begin{array}{c} n_{jk}=\sum_{i=1}^{p}f_{k}\left(d_{ij}\right) \end{array}$$ (10)

$$\begin{array}{c} n_{j}=\sum_{k=1}^{m}n_{jk} \end{array}$$ (11)

Step 4: Determine the gray evaluation weight vector and matrix

The gray evaluation weight *r*_*jk*_ of the *j*^*th*^ qualitative index for the *k*^*th*^ evaluation grade is calculated using Eq (12) and constitute the gray evaluation weight vector.

$$\begin{array}{c} r_{jk}=\frac{n_{jk}}{n_{j}} \end{array}$$ (12)

All the gray evaluation weight vectors constitute the gray evaluation weight matrix *R*_2_.

#### 3.3.4 Multi-level fuzzy comprehensive evaluation

The fuzzy membership matrix of the quantitative indices *R*_1_ is merged with the gray evaluation weight matrix *R*_2_ to obtain the fuzzy comprehensive evaluation matrix *C*. This matrix *C* is divided into several matrices according to indices in the criterion layer for multi-level fuzzy comprehensive evaluation. The multi-level fuzzy comprehensive evaluation starts from the bottom layer and multiple synthesis operations are then successively carried out upwards until the final results are obtained. Generally, it is divided into a first and second-level fuzzy comprehensive evaluation. The first-level fuzzy comprehensive evaluation comprehensively evaluates each index in the indicator layer, and the second-level fuzzy comprehensive evaluation considers the comprehensive influences between this and the criterion layer.

Step 1: First-level fuzzy comprehensive evaluation

The weight vector of all indices in the indicator layer belonging to the *s*^*th*^ criterion layer is recorded as *W*_*s*_, and the fuzzy comprehensive evaluation matrix of the corresponding indices in the indicator layer is recorded as *C*_*s*_. The first-level fuzzy comprehensive evaluation result *B*_*s*_ is calculated using Eq (13).

$$\begin{array}{c} B_{s}=W_{s}\cdot C_{s} \end{array}$$ (13)

Step 2: Second-level fuzzy comprehensive evaluation

The second-level fuzzy comprehensive evaluation matrix, *D*, is composed of the first-level fuzzy comprehensive evaluation result, *B*_*s*_, in order of rows.

According to the weight vector, *W*, of the indices in the criterion layer and the second-level fuzzy comprehensive evaluation matrix, *D*, the second-level fuzzy comprehensive evaluation result, *B*, is obtained using Eq (14).

$$\begin{array}{c} B=W\cdot D \end{array}$$ (14)

The final evaluation grade can be obtained based on the maximum membership principle.

Step 3: Comprehensive evaluation calculation

The final evaluation value, *Y*, is calculated by the quantified vector for each evaluation gray class, *G*, and the second-level fuzzy comprehensive evaluation result, *B*, using Eq (15).

$$\begin{array}{c} Y=B\cdot G^{T}=B\cdot V^{T} \end{array}$$ (15)

## 4 Case analysis

### 4.1 Project information

Take the capacity expansion project of the Huangling-Yan’an Expressway as an example. The Huangling-Yan’an Expressway is 143.2 km long, with 12 bridges and 22 tunnels. The expressway was designed according to the technical standards for four-lane expressways in hilly terrain.

### 4.2 Quantitative indices score of the Huangling-Yan’an Expressway

Referring to the *Special evaluation report of green highway theme project in Huangling-Yan’an Expressway construction* (hereinafter called *Project Evaluation Report*), the completion performance of quantitative indices of Huangling-Yan’an Expressway were obtained, as shown in Table 6.

**Table 6:** Completion performance of quantitative indices.

Quantitative indices in the indicator layer	Value	Score
Rate of reduction in energy consumption during construction *U*_11_	42.7%	10
Rate of reduction in CO_2_ emissions during construction *U*_12_	62.1%	10
Rate of utilization in durable pavement structure *U*_21_	100%	10
Rate of utilization in warm mix asphalt pavement *U*_22_	26.4%	10
Rate of utilization in high performance concrete *U*_23_	43.2%	8.64
Rate of utilization in recyclable materials *U*_24_	46.1%	9.02
Rate of utilization in renewable energy for building facilities *U*_25_	77.8%	10
Coverage rate of ETC *U*_26_	100%	10

### 4.3 Weights calculation

According to the weight calculation method in Section 3.2, the judgment matrix is obtained, and the weight of each index is calculated after passing the consistency test.

(1) The weights of indices in criterion layer

The judgment matrix, consistency test results and weight calculation results of indices in criterion layer in the green and low-carbon expressway evaluation system are shown in Table 7.

**Table 7:** Completion performance of quantitative indices.

	*U*_1_	*U*_2_	*U*_3_	*U*_4_	Weights
*U*_1_	1	1/7	1	5	0.134
*U*_2_	7	1	7	9	0.692
*U*_3_	1	1/7	1	5	0.134
*U*_4_	1/5	1/9	1/5	1	0.040
Consistency test	λ_*max*_ = 4.2389, *CI* = 0.0796, *RI* = 0.90 *CR* = 0.088 ≤ 0.1 Passes the test				

The weight vector *W* = [0.134, 0.692, 0.134, 0.040].

According to the weight vector, the weights of each index in the criterion layer are sorted by size as: systematic index *U*_2_ > intensity index *U*_1_ = guarantee index *U*_3_ > characteristic index *U*_4_.

(2) The weights of indices in the indicator layer

The indices in the indicator layer corresponding to the intensity indices, systematic indices, guarantee indices and characteristic indices in criterion layer are calculated similarly. The weights of all indices are shown in Table 8.

**Table 8:** The weights of green and low-carbon expressway construction evaluation index system of Huangling-Yan’an Expressway.

Indices in criterion layer	Weight	Indices in indicator layer	Index property	Weight
Intensity index *U*_1_	0.134	Rate of reduction in energy consumption during construction *U*_11_	Quantitative	0.500
		Rate of reduction in CO2 emissions during construction *U*_12_	Quantitative	0.500
Systematic index *U*_2_	0.692	Rate of utilization in durable pavement structure *U*_21_	Quantitative	0.256
		Rate of utilization in warm mix asphalt pavement *U*_22_	Quantitative	0.123
		Rate of utilization in high performance concrete *U*_23_	Quantitative	0.123
		Rate of utilization in recyclable materials *U*_24_	Quantitative	0.256
		Rate of utilization in renewable energy for building facilities *U*_25_	Quantitative	0.048
		Coverage rate of ETC *U*_26_	Quantitative	0.023
		Green building facilities construction *U*_27_	Qualitative	0.048
		Application of public service and low-carbon operation system *U*_28_	Qualitative	0.123
Guarantee index *U*_3_	0.134	Energy conservation and emission reduction organization and working mechanism *U*_31_	Qualitative	0.076
		Statistical monitoring system for energy conservation and emission reduction *U*_32_	Qualitative	0.308
		Evaluation and assessment system for responsibility of energy conservation and emission reduction target *U*_33_	Qualitative	0.308
		Energy conservation and emission reduction publicity and training *U*_34_	Qualitative	0.308
Characteristic index *U*_4_	0.040	Site selection for bridges and tunnels *U*_41_	Qualitative	0.550
		Construction technology for bridges and tunnels *U*_42_	Qualitative	0.214
		Application of tunnel energy-saving lighting technology *U*_43_	Qualitative	0.142
		Application of intelligent control system for tunnel ventilation *U*_44_	Qualitative	0.094

### 4.4 Gray fuzzy comprehensive evaluation

#### 4.4.1 Fuzzy membership of quantitative indices

The comment set is set as {Poor, Eligible, Intermediate, Good, Excellent}, and the value range for each evaluation grade is shown in Table 9. The center point of each value range is taken to obtain the quantitative criteria for each evaluation grade *V* = {*v*_1_, *v*_2_, *v*_3_, *v*_4_, *v*_5_} = {3, 6.5, 7.5, 8.5, 9.5}. According to the method in Section 3.3.1, the fuzzy membership of the quantitative indices in the indicator layer is obtained and composes the membership matrix *R*_1_.

$$\begin{array}{c} R_{1}=[\begin{array}{cccccc} U_{11} & 0 & 0 & 0 & 0 & 1\\ U_{12} & 0 & 0 & 0 & 0 & 1\\ U_{21} & 0 & 0 & 0 & 0 & 1\\ U_{22} & 0 & 0 & 0 & 0 & 1\\ U_{23} & 0 & 0 & 0 & 0.86 & 0.14\\ U_{24} & 0 & 0 & 0 & 0.48 & 0.52\\ U_{25} & 0 & 0 & 0 & 0 & 1\\ U_{26} & 0 & 0 & 0 & 0 & 1 \end{array}] \end{array}$$

**Table 9:** The value range for each evaluation grade.

Evaluation grade	Poor	Eligible	Intermediate	Good	Excellent
Value range	[0, 6]	(6, 7]	(7, 8]	(8, 9]	(9, 10]

#### 4.4.2 Gray evaluation weight of qualitative indices

Step 1: Establish the evaluation sample matrix

An expert group composed of five experts from the Haungling-Yan’an Expressway project evaluated and scored (with 0.5 points as the minimum unit of score) the qualitative indices in the indicator layer of the green and low-carbon expressway and the evaluation sample matrix, *H*, was obtained.

$$\begin{array}{c} H=[\begin{array}{cccccccccc} U_{27} & U_{28} & U_{31} & U_{32} & U_{33} & U_{34} & U_{41} & U_{42} & U_{43} & U_{44}\\ 9.5 & 9 & 8.5 & 9 & 8 & 9 & 9.5 & 8.5 & 9 & 8.5\\ 8 & 7.5 & 8.5 & 9 & 8.5 & 8 & 9 & 9 & 9 & 9\\ 9 & 9 & 8 & 9.5 & 9 & 8.5 & 9.5 & 8.5 & 8.5 & 9.5\\ 8.5 & 8 & 9 & 9 & 8.5 & 9 & 9.5 & 9.5 & 8 & 9\\ 9 & 9 & 8.5 & 9.5 & 9 & 9 & 9.5 & 9 & 8.5 & 8 \end{array}] \end{array}$$

Step 2: Calculate the gray evaluation coefficient Take the evaluation index *U*_27_ as an example:

$$n_{11}=\sum_{i=1}^{5}f_{1}\left(d_{i1}\right)=f_{1}\left(9.5\right)+f_{1}\left(8\right)+f_{1}\left(9\right)+f_{1}\left(8.5\right)+f_{1}\left(9\right)=0$$

Other gray evaluation coefficients for evaluation index *U*_27_ can be calculated similarly:

$$n_{12}=0,\;n_{13}=0.5,\;n_{14}=2.5,\;n_{15}=2$$

The total gray evaluation coefficient:

$$n_{1}=\sum_{j=1}^{5}n_{1j}=5$$

Step 3: Calculate the gray evaluation weight vector and matrix

$$\begin{array}{c} r_{11}=\frac{n_{11}}{n_{1}}=\frac{0}{5}=0;\;r_{12}=\frac{n_{12}}{n_{1}}=\frac{0}{5}=0;\;r_{13}=\frac{n_{13}}{n_{1}}=\frac{0.5}{5}=0.1;\\ r_{14}=\frac{n_{14}}{n_{1}}=\frac{2.5}{5}=0.5;\;r_{15}=\frac{n_{15}}{n_{1}}=\frac{2.0}{5}=0.4 \end{array}$$

The gray evaluation weight vector for evaluation index *U*_27_ can be expressed as:

$$r_{1}=\left(r_{11},r_{12},r_{13},r_{14},r_{15}\right)=\left(0,0,0.1,0.5,0.4\right)$$

Other gray evaluation weight vectors for qualitative evaluation indices in the indicator layer can be calculated similarly, and the gray evaluation weight matrix, *R*_2_, was obtained by summarizing the vectors.

$$\begin{array}{c} R_{2}=[\begin{array}{cccccc} U_{27} & 0 & 0 & 0.1 & 0.5 & 0.4\\ U_{28} & 0 & 0 & 0.3 & 0.4 & 0.3\\ U_{31} & 0 & 0 & 0.1 & 0.8 & 0.1\\ U_{32} & 0 & 0 & 0 & 0.3 & 0.7\\ U_{33} & 0 & 0 & 0.1 & 0.7 & 0.2\\ U_{34} & 0 & 0 & 0.1 & 0.6 & 0.3\\ U_{41} & 0 & 0 & 0 & 0.1 & 0.9\\ U_{42} & 0 & 0 & 0 & 0.6 & 0.4\\ U_{43} & 0 & 0 & 0.1 & 0.7 & 0.2\\ U_{44} & 0 & 0 & 0.1 & 0.5 & 0.4 \end{array}] \end{array}$$

#### 4.4.3 Multi-level fuzzy comprehensive evaluation of green and low-carbon expressway construction

*R*_1_ and *R*_2_ were merged to obtain the fuzzy comprehensive evaluation matrix, *C*, and divided it into *C*_1_, *C*_2_, *C*_3_ and *C*_4_ according to the indices in criteria layer for multi-level fuzzy comprehensive evaluation.

Step 1: First-level fuzzy comprehensive evaluation

$$B_{1}=W_{1}\cdot C_{1}=\left[0.5,0.5\right]\cdot [\begin{array}{ccccc} 0 & 0 & 0 & 0 & 1\\ 0 & 0 & 0 & 0 & 1 \end{array}]=\left[0,0,0,0,1\right]$$

$$B_{2}=W_{2}\cdot C_{2}=\left[0,0,0.0417,0.3019,0.6564\right]$$

$$B_{3}=W_{3}\cdot C_{3}=\left[0,0,0.0692,0.5536,0.3772\right]$$

$$B_{4}=W_{4}\cdot C_{4}=\left[0,0,0.0236,0.3298,0.6466\right]$$

Step 2: Second-level fuzzy comprehensive evaluation The second-level fuzzy comprehensive evaluation matrix, *D*, is composed of *B*_1_, *B*_2_, *B*_3_ and *B*_4_, and the second-level fuzzy comprehensive evaluation result, *B*, is obtained using Eq (14).

$$\begin{array}{c} B=W\cdot D=\left[0.134,0.692,0.134,0.040\right]\cdot [\begin{array}{ccccc} 0 & 0 & 0 & 0 & 1\\ 0 & 0 & 0.0417 & 0.3019 & 0.6564\\ 0 & 0 & 0.0692 & 0.5536 & 0.3772\\ 0 & 0 & 0.0236 & 0.3298 & 0.6466 \end{array}]=[0,0,0.0391,0.2963,0.6646] \end{array}$$

The maximum membership of the evaluation result B is 0.6646 at grade “Excellent”, which is higher than other grades, so the evaluation grade of Huangling-Yan’an Expressway green and low-carbon construction is “Excellent” according to the maximum membership principle.

Step 3: Comprehensive evaluation calculation

The final evaluation value, *Y*, is calculated using Eq (15).

$$Y=B\cdot G^{T}=9.1255$$

This value is a quantitative embodiment of the evaluation grade “Excellent” and can be used to compare the green and low-carbon construction levels of different expressways.

### 4.5 Discussions

1. The evaluation score in the *Project Evaluation Report* is 97.37 according to the *Standard*, which is consistent with the evaluation results where the evaluation grade is “Excellent”, and the evaluation value is 9.1255. This demonstrates the accuracy and feasibility of the proposed evaluation index system and evaluation method. Furthermore, the quantitative processing and score calculation method of evaluation indices are more scientific and specific than those in *Standard*, and the merging of fuzzy membership and gray evaluation weight can effectively combine the quantitative and qualitative indices for a comprehensive evaluation.
2. The proposed evaluation index system and evaluation method can evaluate and analyze the four indices in the criterion layer to find out the weak parts of the project and strengthen the precise management of the project. According to the first-level fuzzy comprehensive evaluation results (*B*_1_, *B*_2_, *B*_3_, and *B*_4_) and maximum membership principle, the Huangling-Yan’an Expressway has reached the “Excellent” grade in terms of intensity index, systematic index, and characteristic index, and “Good” grade in guarantee index, indicating that the publicity and training on energy conservation and emission reduction is the weakness of the project, and it is recommended to strengthen the management of this aspect.
3. Compared with the traditional expert scoring method [13, 15], the more quantitative indices and the gray evaluation weight used for further processing of the experts’ scoring make the evaluation results in this paper more objective. Compared with the evaluation process in literature [14], the evaluation index system in this paper can directly increase or decrease the indices without additional processing.

## 5 Conclusions

### 5.1 Main findings

1. The proposed evaluation index system is divided into the goal layer, the criterion layer, and the indicator layer, with clear levels. The accuracy and feasibility of the evaluation system is demonstrated through the empirical analysis of the Huangling-Yan’an Expressway.
2. The proposed evaluation method in this paper can improve the defects that the AHP method is susceptible to the misjudgment of some experts, and quantitative and qualitative indices cannot be comprehensively evaluated in traditional evaluation methods. The evaluation process and results are intuitive and clear.

### 5.2 Theoretical implications

1. The concept of green roads has gradually been developed and refined from the initial sustainability of the whole life cycle of highway. The proposed evaluation index system focuses on the construction period of the expressway and introduces bridge and tunnel indices as characteristic indices to adapt to the multi-bridge and tunnel characteristics of expressways, especially expressways in mountain regions, which will contribute to the expansion of the concept of green expressway to some purpose.
2. When calculating the weight, an improved AHP method is proposed, which combines the opinions of multiple experts to determine the weight and effectively avoid the situation of inaccurate weight calculation caused by misjudgments of some experts.
3. The fusion of quantitative and qualitative indices can be realized by setting the fuzzy membership function and the whitening weight function as the same function, so that the comprehensive evaluation of all indices can be carried out.

### 5.3 Practical implications

1. The proposed evaluation index system has strong versatility and scalability. It is suitable for green and low-carbon evaluation of expressways and highways with different geomorphic features when increasing or decreasing the characteristic indices.
2. The weight of each index can provide management recommendations to the project management department. Among the four indices in criterion layer in this paper, the systematic index mainly including the use of recyclable and environmentally friendly new materials has the highest weight. It is recommended that the project management department pay special attention to the rational utilization of environmentally friendly materials while paying attention to energy conservation and emission reduction, and actively introduce new materials, which is of positive significance for the green and low-carbon construction of expressways.

### 5.4 Future work

Referring to the idea of the establishment of the proposed evaluation index system, the green and low-carbon evaluation indices of expressway design, operation and management period can be established, and the evaluation of the whole life cycle of expressways with different geomorphic features can be realized by introducing characteristic indices.
